# Transcranial Magnetic Stimulation-Induced Plasticity Mechanisms: TMS-Related Gene Expression and Morphology Changes in a Human Neuron-Like Cell Model

**DOI:** 10.3389/fnmol.2020.528396

**Published:** 2020-10-19

**Authors:** Alix C. Thomson, Gunter Kenis, Sylvia Tielens, Tom A. de Graaf, Teresa Schuhmann, Bart P.F. Rutten, Alexander T. Sack

**Affiliations:** ^1^Department of Cognitive Neuroscience, Faculty of Psychology and Neuroscience, Maastricht University, Maastricht, Netherlands; ^2^School for Mental Health and Neuroscience (MHeNS), Department of Psychiatry and Neuropsychology, Faculty of Health, Medicine and Life Sciences, Maastricht University, Maastricht, Netherlands; ^3^Maastricht Brain Imaging Centre (MBIC), Maastricht University, Maastricht, Netherlands; ^4^Center for Integrative Neuroscience, Faculty of Psychology and Neuroscience, Faculty of Health, Medicine and Life Sciences, Maastricht University, Maastricht, Netherlands

**Keywords:** brain Stimulation, cortical excitability, long term potentiation (LTP), gene expression, SH-SY5Y cells, theta burst stimulation (TBS)

## Abstract

Transcranial Magnetic Stimulation (TMS) is a form of non-invasive brain stimulation, used to alter cortical excitability both in research and clinical applications. The *intermittent* and *continuous* Theta Burst Stimulation (iTBS and cTBS) protocols have been shown to induce opposite after-effects on human cortex excitability. Animal studies have implicated synaptic plasticity mechanisms long-term potentiation (LTP, for iTBS) and depression (LTD, for cTBS). However, the neural basis of TMS effects has not yet been studied in human neuronal cells, in particular at the level of gene expression and synaptogenesis. To investigate responses to TBS in living *human* neurons, we differentiated human SH-SY5Y cells toward a mature neural phenotype, and stimulated them with iTBS, cTBS, or sham (placebo) TBS. Changes in (a) mRNA expression of a set of target genes (previously associated with synaptic plasticity), and (b) morphological parameters of neurite outgrowth following TBS were quantified. We found no general effects of stimulation condition or time on gene expression, though we did observe a significantly enhanced expression of plasticity genes *NTRK2* and *MAPK9* 24 h after iTBS as compared to sham TBS. This specific effect provides unique support for the widely assumed plasticity mechanisms underlying iTBS effects on human cortex excitability. In addition to this protocol-specific increase in plasticity gene expression 24 h after iTBS stimulation, we establish the feasibility of stimulating living human neuron with TBS, and the importance of moving to more complex human *in vitro* models to understand the underlying plasticity mechanisms of TBS stimulation.

## Introduction

Transcranial Magnetic Stimulation (TMS) is a widely used neuromodulation technique, where electromagnetic pulses can non-invasively stimulate cortical structures (Barker et al., 1985; Hallett, 2007). Multiple pulses administered in a certain frequency (repetitive TMS: rTMS), can have effects on cortical excitability lasting beyond the period of stimulation (Pascual-Leone et al., 1994; Huang et al., 2005). In humans, such effects are often revealed with physiological outcome measures, such as Motor-Evoked Potentials (MEPs; Rothwell et al., 1999). For example, the commonly used Theta Burst Stimulation (TBS) protocols *intermittent* and *continuous* TBS (iTBS and cTBS) have been shown to increase or decrease MEPs for up to 1 h following stimulation, respectively, (Huang et al., 2005). Still, large inter and intra subject variability have been associated with the use of MEP’s as an outcome measure (Schilberg et al., 2017). Several reports on the difficulty of replicating the assumed iTBS/cTBS effects have cast doubt on the efficacy of these protocols (Hamada et al., 2013; Lopez-Alonso et al., 2014; Tse et al., 2018; Thomson et al., 2019). A method to reliably verify rTMS effects, for example in an *in vivo* model, is urgently needed.

A widespread assumption is that such after-effects are attributable to neuronal plasticity mechanisms, such as long-term potentiation (LTP) and long-term depression (LTD) (Suppa et al., 2016). Indeed, administering an N-methyl-D-aspartate receptor (NMDAR) antagonist to participants prior to iTBS/cTBS stimulation has been shown to completely abolish the after-effects on MEP amplitude, relating NMDAR-dependent LTP/LTD to TBS effects in humans (Huang et al., 2007).

Long-term potentiation is a well-studied form of synaptic plasticity, often induced *ex vivo* through high frequency stimulation directly to individual neurons or groups of neurons (Bliss and Lomo, 1973). It can be divided into two phases, early-LTP, which is protein-synthesis independent, and occurs immediately after stimulation, and late-LTP, which requires protein synthesis and can lead to structural and functional changes lasting at least 24 h *in vitro* (Frey et al., 1993; Abel et al., 1997; Kandel, 2001; Baltaci et al., 2019). The phenomenon of late LTP depends heavily on brain-derived neurotrophic factor (BDNF) binding to its high affinity receptor, tyrosine kinase receptor B (TrkB), and initiating a series of signaling proteins leading to changes in expression of plasticity related genes (Matsuki, 1998; Sermasi and Domenici, 1998; Gottschalk et al., 1999; Huang et al., 1999).

In cultured mouse neurons, rTMS has been shown to activate this BDNF-TrkB signaling pathway (Muller et al., 2011; Wang et al., 2011; Ma et al., 2013), as well as to induce an immediate release of calcium from intracellular stores (Grehl et al., 2015; Banerjee et al., 2017), which is important in the induction of synaptic plasticity (Hulme et al., 2012). In rats, high and low frequency rTMS stimulation showed differential activation of the immediate early genes *C-FOS* (a general marker for excitatory cell activity) and *EGR1* (a presumed marker of LTP or LTD induction) (Aydin-Abidin et al., 2008). In addition, iTBS and cTBS showed dose-dependent and protocol specific effects on the synthesis of these two proteins (Volz et al., 2013). iTBS and cTBS also differentially change the synthesis of calcium binding proteins in rats. The latter is related to modulation of inhibitory interneurons (Trippe et al., 2009; Benali et al., 2011; Mix et al., 2014, 2015), which has a functional impact on neuronal electrical activity, with iTBS, but not cTBS, enhancing spontaneous neuronal firing and EEG gamma band power (Benali et al., 2011).

In sum, several lines of cellular evidence from animal studies implicate different aspects of late LTP mechanisms in the after-effects of rTMS. But such plasticity effects of rTMS at the cellular and molecular level have mainly been examined in rodent-based models. Given the rapid development and increasingly widespread and accepted use of rTMS, particularly TBS, for both experimental and clinical applications in human volunteers and patients, it seems crucial to study and understand the cellular effects of TBS in human neurons. *In vitro* studies with human neurons could validate the animal results, and contribute to our understanding of the mechanisms of action underlying different TBS protocols that are already, and increasingly, (clinically) applied worldwide.

To our knowledge, only two previous studies have used human SH-SY5Y neuroblastoma cells to measure responses to rTMS *in vitro*, and both used classical high and low frequency protocols. One study reported protocol-specific effects of high (9 Hz) and low (3 Hz) frequency rTMS stimulation on catecholamine levels and neurotransmitter metabolism (Shaul et al., 2003), and the other showed increased intracellular cAMP and CREB activation with high (5 Hz) frequency rTMS (Hellmann et al., 2012). These studies provide the first evidence of the feasibility of using SH-SY5Y cells in this type of study. However, to date no study has used SH-SY5Y cells to investigate neural responses to TBS protocols.

Here, we developed an *in vitro* human neuron model to assess protocol-specific effects of iTBS/cTBS on plasticity markers of gene expression and neurite outgrowth. We chose to investigate changes in the BDNF-TrkB signaling cascade, given its importance in plasticity mechanisms, and because previous animal studies have shown an rTMS-induced effect on protein expression in this pathway (Frey et al., 1993; Gottschalk et al., 1999; Kandel, 2001). We focused on hypothesis-driven gene expression targets in this pathway, to identify immediate effects to help tailor future protein or genome-wide screening analysis. We also wanted to quantify any structural changes to neurite morphology with commonly used cytoskeletal markers βIII-Tubulin and MAP2, which may indicate neuroplastic effects. We differentiated SH-SY5Y cells into a mature neuron-like phenotype, applied different TBS protocols, and collected cells immediately, 3 h, 6 h, and 24 h after stimulation. While in humans TBS effects have been shown to be strongest up to 30 min after stimulation (Huang et al., 2005), we chose these time points to capture the plasticity-dependent processes requiring longer periods of time (Dent et al., 1999; Minichiello, 2009; Yap and Greenberg, 2018).

We report a protocol-specific effect on expression of genes in the BDNF-TrkB pathway, with an increase in expression of *NTRK2* and *MAPK9* 24 h after iTBS stimulation, but no change in cell count, neurite length, neurite branching, or levels of neurite proteins. In a separate report, we showed that these TBS procedures, using the same *in vitro* model, did affect excitability as hypothesized. This suggests that the results reported here did originate from functionally active cell cultures, that responded to TBS as hypothesized (Thomson et al., 2020). The current results, positive as well as negative, thus demonstrate the feasibility and value of *in vitro* human neuron studies to unravel plasticity mechanisms induced by TMS.

## Methods

### Cell Culture

SH-SY5Y cells were obtained from ATCC^®^ (Cat #CRL2266^TM^, RRID:CVCL_0019) and were maintained and expanded according to ATCC^®^ recommendations. For experiments, cells were not used above passage 26. Cells were grown in DMEM/Nut Mix F12 with Glut-L (Gibco^TM^, Thermo Scientific) supplemented with 10% heat inactivated fetal bovine serum (FBS, MERCK), 1% penicillin-streptomycin (P/S), and 1% L-Glutamate at 37°C and 5% C0_2_. Experiments were conducted on differentiated cells plated in round 35 mm dishes at approximately 2.4 × 10^4^ cells per well. Differentiation was induced over a period of 13 days; FBS supplementation was decreased to 3% 3 days prior to the addition of 10 μM retinoic acid for 10 days (RA; Sigma-Aldrich, R2625). Medium was replaced every 2 days.

### Magnetic Stimulation

**FIGURE 1:**
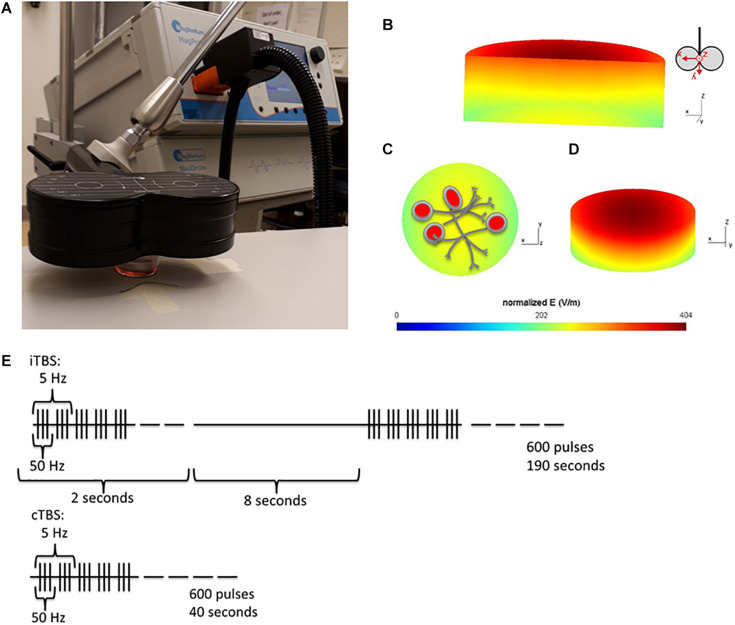
Experiment Setup. **(A)** Position of the cell culture dish 1cm below the center of the coil. **(B–D)** Simulation of the induced electric field (V/m) within the cell culture dish. SimNIBS (Thielscher et al., 2015) was used to calculate the electric field induced within the cell culture dish, during TMS stimulation at 100% MSO. The simulation parameters (cell culture dish model and conductivity values) were generously shared by Lenz et al. (2016). **(B)** A cross section of the cell culture dish, showing the gradient of induced electric field within the dish. The electric field is strongest at the top of the dish, closest to the coil. Coil orientation shown beside. **(C)** Shows the bottom surface of the cell culture dish (furthest away from the coil, where the cells are plated) and **(D)** Is a tilted view of the dish from the top surface. **(E)** Stimulation protocols used for stimulation, iTBS has been shown to increase cortical excitability (measured in Motor evoked potentials), and cTBS to decrease it for up to 1 h following stimulation (Huang et al., 2005).

Cells were placed 1 cm below the center of a Cool-B65 figure of 8 coil (Magventure, Denmark) and stimulated at 100% Maximum Stimulator Output (MSO) with a MagPro X100 with MagOption stimulator, realized output 143 A/μS (Magventure, Denmark). The setup is illustrated in Figure 1A. Each stimulation consisted of the Huang et al. (2005) published protocol of 50 Hz triplets repeated in a 5 Hz rhythm. cTBS was a continuous train, while iTBS was a 2 s train of pulses, with an inter-train interval of 8 s, both for 600 pulses (Huang et al., 2005).

Cells stimulated with cTBS remained under the coil for an additional 150 s, and cells in the sham condition were placed under the coil for 190 s, to ensure that cells in all TBS conditions (cTBS, iTBS, and sham) were out of the incubator for the same amount of time. The electrical field induced in the dish, with the stimulation conditions described above (100% MSO, dish placed 1 cm below the coil), was simulated using the SimNIBS toolbox (Thielscher et al., 2015). The cell culture dish mesh was generously shared by the authors of (Lenz et al., 2016). The distribution of the electric field (V/m) within the dish from several viewpoints can be seen in Figures 1B–D. The stimulation protocol is shown in Figure 1E.

### qRT-PCR

Cells were collected immediately, 6 h or 24 h after stimulation. In humans, the maximal TBS effects are expected in the first 30 min after stimulation (Huang et al., 2005). However, we chose to measure at later time points because we were specifically interested in plasticity-dependent gene expression, which require hours or even days (Minichiello, 2009). While the rapid expression of immediate early genes could be effected by TBS within the first 30 min (Abraham et al., 1991; Jones et al., 2001), most of the genes of interest in our study are expressed at later time points following plasticity-inducing protocols (Yap and Greenberg, 2018). RNA was extracted with TRIzol (Invitrogen, 15596026) according to the manufacturer’s instructions. Nanodrop was used to quantify the amount of RNA in each sample, and cDNA was synthesized using RevertAid H Minus First Strand cDNA Synthesis Kit (Thermo Scientific, K1632). RNA was stored at −80°C, cDNA at −20°C. Eight biological replicates were collected per stimulation condition per time point, derived from at least two undifferentiated cell batches for differentiation. Due to quality of extracted RNA, some samples had to be discarded, leaving between four and eight biological replicates per condition. Each biological replicate was run in technical duplicates for qRT-PCR. A complete list of biological replicates and differentiation batches for each sample can be found in the Supplementary Material (Supplementary Table 1).

Primers for qPCR were designed using the NCBI gene reference database and Primer-BLAST (National Library of Medicine). The following genes were analyzed (see Supplementary Table 2 for sequences): *NTRK2, BCL2, MAPK9*, *TUBB3*, *EGR1*, *CREB1*, and *GAPDH*, *PPiB*, and *TBP* were used as housekeeping genes (HKGs). Primers, 600 nM, were mixed with Fast Start Universal Sybr green ROX (Roche, 491385001). Samples were run in 384 well qPCR plates (Roche, 4TI-0382) using the LightCycler^®^ 480 Real-Time PCR system (Roche LifeScience). qPCR program details are described in Supplementary Material (Supplementary Table 3).

### Microscopy

Cells were grown on 12 mm round glass cover slips (VWR, 631-1577), coated with 1 μg/mL Laminin (Sigma, L2020) and 100 μg/mL Poly-L-Ornithine (Sigma, P4957), and cultured as described above.

Fluorescence microscopy was used to visualize morphological changes 3, 6, and 24 h after stimulation. Again, this is because these structural changes require longer time windows to visualize effects. Axonal reorganization has been shown to require several hours (Pascual-Leone et al., 1994; Rothwell et al., 1999; Huang et al., 2005; Hallett, 2007; Schilberg et al., 2017) to begin to show signs of microtubule movement (Dent et al., 1999). Cells were washed in PBS and fixed for 10 min at room temperature in 4% paraformaldehyde. Fixed and PBS-washed cells were blocked in PBS-T (PBS + 0.1% Tween-20) and 10% donkey serum followed by primary antibody incubation. Antibodies for marking neurite outgrowth [βIII-Tubulin (Cell Signaling, Cat #5568S, RRID:AB_10694505)] and axons [MAP2 (Sigma, Cat #M2320, AB_609904)] were used. Cells were washed in alternating PBS-T and PBS, and incubated with secondary antibodies donkey-anti-rabbit Alexa 488 (Invitrogen, Cat #A-21206 RRID:AB_141708), donkey-anti-mouse Alexa 594 (Invitrogen, Cat #A-21203, RRID:AB_141633), and with DAPI (CarlRoth, Cat #6843.3). The glass cover slips were then mounted on glass microscope slides and imaged with an Olympus BX51WI microscope and Disk Spinning Unit. Pictures were taken using the 20X objective lens. Further details on primary and secondary antibodies and microscope settings are listed in the Supplementary Material (Supplementary Tables 4, 5). This experiment was repeated twice, with 4 images of each replicate analyzed. In total, 8 images per stimulation condition time point were included; with each image containing on average 163 ± 73 cells.

### Analysis

#### Gene Expression

A standard curve was used to calculate relative concentrations of gene expression per gene. An average of technical duplicates was made, and normalized to the average of the 3 HKGs (GAPDH, PPiB, and TBP). Data were analyzed with LightCycler 480 software version 1.5.1.62 (Roche Life Sciences) and Microsoft Excel, and graphs were made in Prism 5 (GraphPad Software, United States, RRID: SCR_002798).

#### Microscopy

Images were processed and analyzed with Fiji (ImageJ version 1.52i, RRID:SCR_002285; Schindelin et al., 2012). Cells in each picture were counted with the analyze particle tool, using the DAPI stain for the cell nuclei. Fluorescence intensity (immunoreactivity of βIII-Tubulin) was quantified by measuring the total 488 channel intensity in each image. This was then divided by the total fluorescence intensity in the 350 channel, to give a corrected fluorescence for the number of cells in the image. Neurite length and branching were quantified by tracing outgrowths in the 488 (βIII-Tubulin) channel. Neurite length was measured with the segmented line tool, at 20x magnification and quantifying 20 cells per image. From each cell only the primary neurite length was counted. The NeuronJ plugin (Meijering et al., 2004) was used to quantify neurite branching. For each image, all neurites were semi-automatically traced, and manually labeled as either primary, secondary, or tertiary extensions. The number of branches (secondary or tertiary extensions) were divided by the total number of neurons (counted with DAPI), to give the number of branches per neuron in each image. Graphs were made with Prism 5 (GraphPad Software, United States, RRID:SCR_002798).

### Statistical Analysis

Statistical analysis was done using IBM SPSS 24 (SPSS for Windows version 24.0. Armonk, NY, United States: IBM Corp). SH-SY5Y differentiation was verified through independent samples *t*-tests comparing undifferentiated and differentiated cells. Biological replicates were used for statistical analysis. For gene expression analysis, a 2-way ANOVA was used to first test HKGs for an effect of stimulation condition or time on expression. None of the genes showed any significant effects (complete results in Supplementary Data 1.3). Since there was no significant effect of stimulation condition on expression of any genes at the immediate time point (complete results under Supplementary Data 1.1 and Supplementary Figure 1 in Supplementary Material), these levels were averaged across stimulation conditions, and used to calculate % immediate expression levels for the 6 and 24-h time points.

Due to the small number of biological replicates and unequal variances across samples, non-parametric Kruskal–Wallis testes were done for condition and time separately. However, using these non-parametric statistical tests did not allow for testing of interaction effects. We had expected gene expression effects to be strongest at one of the time points, therefore we performed hypothesis-driven analyses for the 6 h and 24 h time points separately, using the Kruskal–Wallis test for a significant difference in gene expression between stimulation conditions. Reported gene expression and microscopy results are presented as mean ± standard error of the mean. Bonferroni correction was used for *post hoc* comparisons. Figures show bar graphs of the mean, error bars are standard error of the mean.

## Results

### SH-SY5Y Differentiation

**FIGURE 2:**
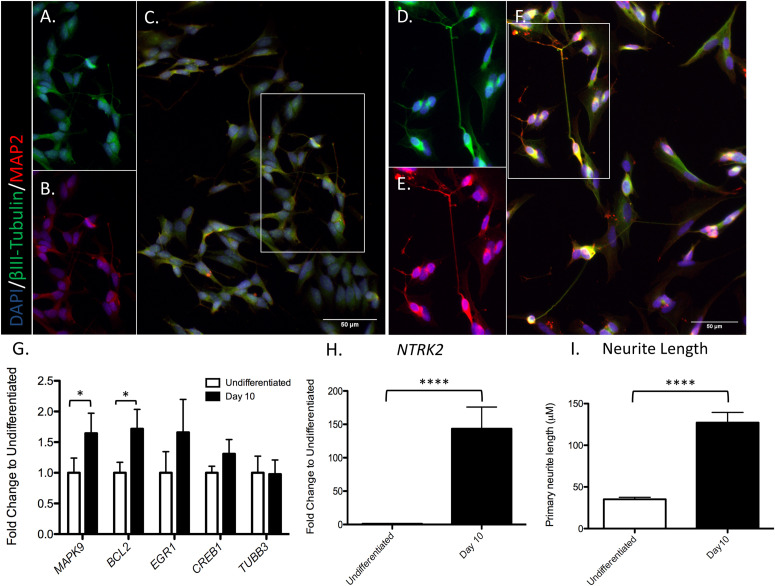
SH-SY5Y cell differentiation. Cells were marked for nucleus DAPI (blue), MAP2 (red), and βIII-Tubulin (green) in an undifferentiated state **(A–C)** or after 10 days of differentiation **(D–F)**. A selection of neurons in the image were chosen to split by channel (βIII-Tubulin or MAP2), identified by the white box in **C** and **F**. **(A)** Selection of undifferentiated cells, βIII-Tubulin. **(B)** Selection of undifferentiated cells, MAP2. **(C)** Full image undifferentiated cells, merge of βIII-Tubulin and MAP2. **(D)** Selection of 10 days differentiated cells, βIII-Tubulin. **(E)** Selection of 10 days differentiated cells, MAP2. **(F)** Full image of 10 days differentiated cells; merge of βIII-Tubulin and MAP2. **(G)** RT-qPCR analysis was used to assess the expression of the indicated genes in differentiated and undifferentiated cells. **(H)** Significant increase in *NTRK2* expression at day 10 of differentiation. **(I)** Significant increase in primary neurite outgrowth at day 10 of differentiation. Values represent mean ± SEM (^∗^*P* < 0.05, ^****^P < 0.001, and Student’s *t*-test).

Differentiation status of SH-SY5Y cells was verified through visual inspection of increased neurite length, and confirmed by a significant increase in TrkB expression (*NTRK2)* at day 10 of differentiation [*t*(7) = 8.657, *p* < 0.0001], as reported previously (Ehrhard et al., 1993; Jahn et al., 2017; see Figure 2). Expression of all genes of interest was verified at day 10 of differentiation (Figures 2G,H). Complete results of *t*-tests are reported in Supplementary Material (Supplementary Data 1.2). Neurite outgrowth increased from day 0 to day 10 of differentiation (37.9 ± 2.8 μm and 112.3 ± 11.8 μm, respectively; *t*(26) = 6.163, *p* < 0.0001; see Figure 2I).

### Effects of Stimulation Condition on Gene Expression

We were interested in gene expression changes in the BDNF-TrkB signaling cascade, specifically in downstream targets related to plasticity. Therefore, we focused on the following genes involved in this pathway: Mitogen-Activated Protein Kinase 9 (*MAPK9*, GeneID: 5601), Neurotrophic Regulator Tyrosine Kinase 2 (*NTRK2*, GeneID: 4915), B-cell lymphoma 2 (*BCL2*, Gene ID: 596), Tubulin Beta Class III (*TUBB3*, Gene ID: 10381), and cAMP Responsive Element Binding Protein 1 (*CREB1*, Gene ID: 1385). We also included Early Growth Response 1 (*EGR1*, Gene ID: 1958), which is considered an immediate early gene.

We found no significant effect of stimulation condition or time on several of the genes tested; *BCL2* [Condition *H*(2) = 0.125, *p* = 0.940, Time *H*(1) = 2.626, *p* = 0.105]; *TUBB3* [Condition *H*(2) = 1.060, *p* = 0.589, Time *H*(1) = 1.298, *p* = 0.255]; and *CREB1* [Condition *H*(2) = 0.651, *p* = 0.722, Time *H*(1) = 0.006, *p* = 0.936].

**FIGURE 3:**
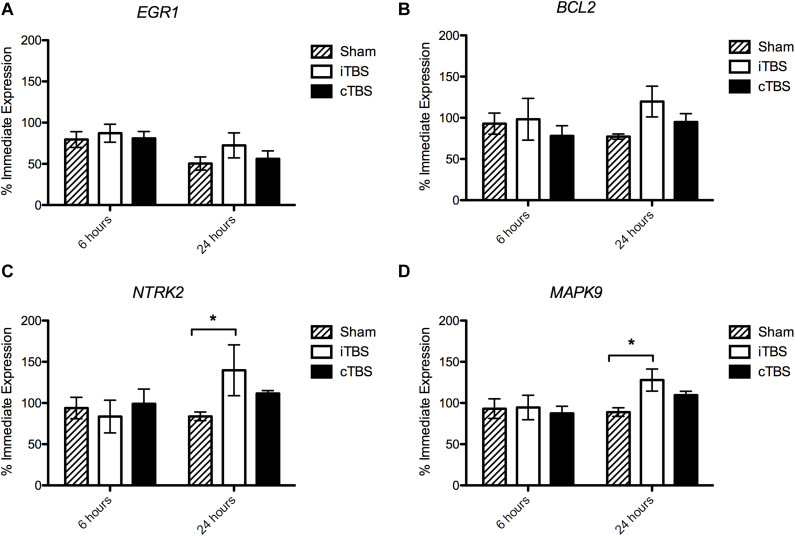
Gene Expression results 6 and 24 hours following stimulation. Values are normalized by the average of 3 Housekeeping Genes (*GAPDH, TBP*, and *PPiB*) and divided by the average immediate expression. Bars shown are % immediate time point expression. **(A)** Expression of *EGR1*. **(B)** Expression of *BCL2*. **(C)** Expression of *NTRK2*. **(D)** Expression of *MAPK9*. Significant bonferroni-corrected *post hoc* tests are indicated with a *(*P* < 0.05), *n* = 4–8, see Supplementary Material for exact replicate numbers per condition.

We found no significant effect of condition on *EGR1* expression [*H*(2) = 1.926, *p* = 0.382], but an effect of time [*H*(1) = 9.195, *p* = 0.002], with a decrease in the expression (% immediate) at 24 h (54.5 ± 5.6%) compared to 6 h (77.4 ± 5.0%; Figure 3A). Similarly for *MAPK9* we find no significant effect of condition [*H*(2) = 1.043, *p* = 0.594) but an effect of time (*H*(1) = 4.152, *p* = 0.042], as well as for *NTRK2* expression [Condition *H*(2) = 0.905, *p* = 0.636, Time *H*(1) = 4.022, *p* = 0.045].

#### BCL2 Expression

There was no effect of stimulation condition at 6 h [*H*(2) = 1.024, *p* = 0.985]. However, at 24 h, we observed a borderline statistically significant effect of stimulation condition on *BCL2* expression [*H*(2) = 5.981, *p* = 0.050]; i.e., we observed an increase in *BCL2* expression in cells which had been stimulated with iTBS (119.8 ± 18.7%) compared to cTBS (95.0 ± 10.1%) and sham (77.2 ± 3.2%), however, none of the *post hoc* comparisons were significant (*p* > 0.05; Figure 3B).

#### NTRK2 Expression

We find no effect of stimulation condition at 6 h [*H*(2) = 2.12, *p* = 0.346]. At 24 h we find a statistically significant effect of stimulation condition on *NTRK2* expression [*H*(2) = 8.010, *p* = 0.018]. We observed an increase in expression in cells which have been iTBS stimulated compared to sham stimulated cells (139.7 ± 30.85% and 83.8 ± 5.2%, respectively; *p* = 0.036; Figure 3C).

#### MAPK9 Expression

Again, we find no effect of stimulation condition at 6 h [*H*(2) = 0.030, *p* = 0.985]. When analyzing the 24 h time point separately, we observed a statistically significant effect of stimulation condition [*H*(2) = 8.640, *p* = 0.013]. *MAPK9* expression levels were significantly higher in iTBS stimulated cells, compared to sham stimulated cells (127.9 ± 13.4% and 89.0 ± 5.3%, respectively; *p* = 0.017; Figure 3D).

### Effect of Stimulation Condition on Neuron Morphology

#### Cell Count

**FIGURE 4:**
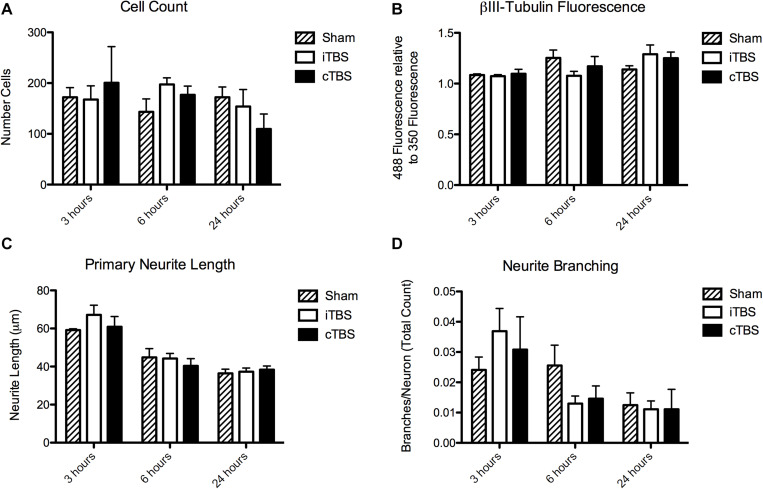
Morphological outcome parameters over time for each stimulation condition. **(A)** Cell count. **(B)** Total fluorescence in the βIII-Tubulin channel (488), normalized to the DAPI channel (350). **(C)** Primary neurite length. **(D)** Neurite branching. *N* = 8 images per stimulation condition.

We found no significant effect of condition [*H*(2) = 0.815, *p* = 0.665] or time [*H*(2) = 3.37, *p* = 0.185] on cell count (Figure 4A).

#### βIII-Tubulin Immunoreactivity

There was no significant effect of stimulation condition on total fluorescence intensity of βIII-Tubulin [*H*(2) = 1.19, *p* = 0.55]. There was, however, a significant effect of time [*H*(2) = 6.61, *p* = 0.037], with an increase at 24 h (1.23 ± 0.05) compared to 6 h (1.17 ± 0.05) and 3 h (1.09 ± 0.007; Figure 4B).

#### Neurite Outgrowth

There was no significant effect of stimulation condition on primary neurite length [*H*(2) = 0.336, *p* = 0.85]. There is a significant effect of time [*H*(2) = 22.320, *p* < 0.001]. Neurites were longer at 3 h (66.56 ± 2.35 μm) compared to 6 h (49.82 ± 2.00 μm) and 24 h (47.92 ± 2.48 μm; Figure 4C).

#### Neurite Branching

There was no significant effect of stimulation condition on neurite branching [*H*(2) = 1.580, *p* = 0.45]. There is a significant effect of time [*H*(2) = 13.901, *p* = 0.001]. There were more branches per neuron at 3 h (0.031 ± 0.0044) compared to at 6 h (0.018 ± 0.0023), and 24 h (0.012 ± 0.0026; Figure 4D).

## Discussion

To date, the molecular and cellular mechanisms underlying TMS have been mainly studied in living rodents or animal brain slices (Suppa et al., 2016; Cirillo et al., 2017). Here, we set out to investigate TMS induced plasticity mechanisms in an *in vitro* human neuron-like model, through stimulating differentiated SH-SY5Y neuroblastoma cells with either iTBS, cTBS, or sham stimulation. Previous animal and human TBS studies have suggested that iTBS/cTBS may rely on activity-dependent plasticity mechanisms of LTP/LTD (Huang et al., 2007; Teo et al., 2007; Wankerl et al., 2010; Hoppenrath and Funke, 2013; Volz et al., 2013). To investigate these changes in living human neurons, we focused on molecular (in particular genes related to BDNF/TrkB signaling) and morphological markers of plasticity. The induced electric field within the cell culture dish was modeled using the SimNIBS toolbox (Thielscher et al., 2015), not with the aim of comparing stimulation strength to that usually achieved with TMS in a human brain, but only to confirm that stimulation was capable of depolarizing neurons. Our high-intensity stimulation parameters were based on other rTMS animal and cell culture studies (Hellmann et al., 2012; Li et al., 2017), with the primary goal of ensuring sufficient depolarization to induce excitations in our cells. Indeed, this was successful, as in another report (Thomson et al., 2020) we could show that these procedures/parameters affected excitability in identically treated cell cultures as hypothesized: demonstrating after-effects in opposite directions for iTBS and cTBS as previously observed in animal and human *in vivo* studies (Suppa et al., 2016).

We found that, compared to sham stimulation, iTBS increased the expression of *NTRK2* and *MAPK9* after 24 h. *MAPK9*, also known as *JNK2*, has been shown in mice to be important in hippocampal synaptic plasticity (Chen et al., 2005; Seo et al., 2012). MAPK9 knockout mice had impaired late but not early LTP, suggesting that *MAPK9* may be instrumental in the switch from early to late LTP (Wankerl et al., 2010). This switch is important, as late LTP is responsible for plasticity effects lasting at least 24 h, requiring protein synthesis and structural changes (Frey et al., 1993; Abel et al., 1997; Yin et al., 2016). In humans, several studies have shown that repeating iTBS (Tse et al., 2018) or cTBS (Goldsworthy et al., 2015) at spaced intervals consolidates LTP/LTD-like effects, providing evidence for late-LTP or late-LTD mechanisms only after repeated iTBS/cTBS sessions. Our results showing an increase in MAPK9 mRNA expression at 24 h following iTBS could indicate this critical shift from early to late LTP mechanisms. Measuring MAPK9 mRNA expression after repeated iTBS of SH-SY5Y cells could further support the evidence from human studies, that repeating iTBS sessions results in late-LTP mechanisms in humans.

Similarly, *NTRK2*, the gene that codes for the high affinity BDNF-receptor TrkB, is thought to be a critical regulator of hippocampal LTP (Minichiello, 2009). Mice lacking TrkB receptors showed reduced TBS-induced LTP, indicating the importance of this receptor in regulating synaptic plasticity (Minichiello et al., 1999). An increase in *NTRK2* mRNA expression indicates that the BDNF-TrkB signaling cascade may be upregulated 24 h after iTBS. This supports the assumption that iTBS promotes LTP-like plasticity, specifically through up-regulation of the BDNF-TrkB pathway.

We also found a slight effect of condition at 24 h following stimulation in expression of *BCL2*. This indicates an effect of TBS on the expression of this gene, but since no *post hoc* comparisons were significant, we cannot conclude that this expression is protocol-specific. This expression of *BCL2* is similar to the expression of *MAPK9* and *NTRK2*, with iTBS stimulated cells showing increased expression compared to sham stimulated cells. *BCL2* is an integral outer mitochondrial membrane protein, and an important regulator of apoptosis (Huang et al., 1998). Its expression is strongly induced by BDNF-TrkB signaling, and has been shown to affect plasticity mechanisms (Licznerski and Jonas, 2018). In other words, the increase in *BCL2* expression that we report may be related to enhanced plasticity and neuroprotective mechanisms 24 h after TBS. More specific apoptosis assays would be required to confirm this. Altogether, our gene expression findings support the hypothesis that the iTBS protocol enhances plasticity mechanisms induced by BDNF-TrkB signaling, confirming evidence from animal experiments. We also found a time effect for the expression of *EGR1*, an important neuronal immediate early gene, functioning as a transcriptional regulator for genes involved in differentiation and neuroplastic changes (Gallo et al., 2018). This increase in *EGR1* expression is critical for the induction of LTP, as an initial increase in *EGR1* expression within 10 min to 2 h after stimulation is required for protein-synthesis dependent late-LTP mechanisms (Abraham et al., 1991; Jones et al., 2001; Koldamova et al., 2014). In other words, an increase in EGR1 expression immediately after stimulation supports TBS-induced plasticity mechanisms.

To examine possible effects of TBS on neuronal morphology, we used immunocytochemistry to visualize neurite outgrowths (βIII-Tubulin) and axons (MAP2). These are widely used as mature, neural cytoskeletal markers in studies of SH-SY5Y cells (Encinas et al., 2000; Christensen et al., 2011; Cui et al., 2017; Jahn et al., 2017; De Simone et al., 2018; Kapalczynska et al., 2018). βIII-Tubulin is an important protein of the microtubule cytoskeleton, expressed primarily in neurons and is critical for axonal guidance and maintenance in mammals (Tischfield et al., 2010). We found, on average 30% (±10%) of neurons expressed βIII-Tubulin, 11.3% (±4.6%) MAP2 and 9.8% (±3.5%) expressed both markers. Representational images of neuron morphology after each stimulation condition, and each time point, can be seen in Supplementary Material (Supplementary Figure 2). Using qPCR as described above, we found no change in the mRNA expression of *TUBB3*, the gene coding for βIII-Tubulin protein, which aligns with our βIII-Tubulin immunoreactivity findings. We did observe a small decrease of axonal length and branches per neuron over time, but without an effect of stimulation condition. This might be related to manipulation of the cell cultures during the stimulation paradigm. Whether TBS induces structural plasticity changes should be further investigated over longer time periods.

In contrast to previous animal studies showing protocol specific changes in plasticity markers following TBS (Trippe et al., 2009; Volz et al., 2013; Labedi et al., 2014), we did not see any effects of cTBS on gene expression or neuron morphology. Additionally, the effects on gene expression that we did see were subtle, and in just two plasticity genes. Importantly, however, the protocol-specific effects reported in these animal studies were found in different cortical areas, therefore it is difficult to compare these results to cell culture which contain a single functional cell type in a single spatial organization. Animal models or slice cultures also contain a functionally relevant organization of different neuron types, such as a mix of inhibitory interneurons and excitatory pyramidal neurons. This neuronal organization might be important, as these studies suggest that iTBS/cTBS is related to differential effects on cortical inhibition (affecting the inhibitory interneurons) (Benali et al., 2011; Hoppenrath and Funke, 2013; Volz et al., 2013). Indeed, as computational modeling has shown, the TMS-induced electric field depends critically on the complex microscopic and macroscopic anatomy of the human cortex (Guidi et al., 1989; Maccabee et al., 1993; Goetz and Deng, 2017). In light of this requirement for complex neuronal organization, our null results become more important, as they might begin to inform us on the minimal level of neuronal organization complexity required for TBS effects on expression of certain genes.

In addition, animal studies often use different stimulation parameters, for example repeating the established Huang et al., 2005 TBS protocol up to five times (Trippe et al., 2009; Volz et al., 2013; Labedi et al., 2014). This greater number of stimulation pulses in these animal studies could also explain why we did not replicate any of the protocol specific changes described in the animal literature. On the other hand, as mentioned, in another set of experiments (Thomson et al., 2020) we did successfully use calcium imaging to reveal the hypothesized TBS effects in our cell cultures, suggesting that our TBS protocols were at least sufficiently strong to induce excitability changes.

We opted to use SH-SY5Y neuroblastoma cells, a human-derived cell line widely used as an *in vitro* model of human neurons. These cells express a variety of neural markers, and can be further differentiated to a more mature neuronal phenotype, having longer neurite outgrowths, increased expression of mature neuron markers, and the formation of mature synapses (Biedler et al., 1978; Encinas et al., 2000; Jahn et al., 2017). Differentiated SH-SY5Y cells have also been shown to produce action potentials (Toselli et al., 1996; Tosetti et al., 1998; Brown et al., 2005; Jahn et al., 2017), and are therefore functionally active neural cells. We have also recently demonstrated TBS protocol-specific functional effects on SH-SY5Y cells using calcium imaging (Thomson et al., 2020). They are a widely used model for a range of research applications such as Parkinson’s disease (Xicoy et al., 2017), pathogenesis of viruses (Christensen et al., 2011), drug efficacy and toxicity (Henkel et al., 2008; Forster et al., 2016; De Simone et al., 2018), and as a 3D cell culture (Cui et al., 2017; Kapalczynska et al., 2018). These cells can also be used in the study of human neuron plasticity and synapse formation (Jahn et al., 2017) for example in the context of examining treatment targets of depression (Leskiewicz et al., 2013; Xu et al., 2019). They are also relatively easy to handle, making them a good candidate to investigate plasticity mechanisms following rTMS.

However, as these cells were derived from malignant tumors (Biedler et al., 1978), cultures may contain two morphologically distant phenotypes, neuroblast-like and epithelial-like (Kovalevich and Langford, 2013). While differentiation protocols aim to establish the most neuron-like phenotype among all cells (Encinas et al., 2000; Shipley et al., 2016; Jahn et al., 2017), there are often inconsistencies among the proportion of phenotypes within each culture. Experimental conditions may also influence the consistency of differentiation or cellular phenotypes in our cultures. For example, removing the cells from the incubator for stimulation, and having a prolonged incubation for the 24-h time point may have contributed to the time effects seen in the *EGR1* expression, primary neurite length, and βIII-Tubulin fluorescence intensity.

We chose to measure changes in *gene expression*, specifically in the BDNF-TrkB signaling cascade, shown to be important in LTP-dependent plasticity mechanisms (Minichiello et al., 1999; Minichiello, 2009, 2014). However, investigation of relevant changes at the protein level following stimulation are also important. For example, future studies could expand on our findings by focusing on protein phosphorylation in the BDNF-TrkB signaling cascade, or investigating whether these plasticity mechanisms are NMDA-receptor dependent. We have taken first steps toward investigating TBS-induced changes in human neurons *in vitro*, but more studies are needed to better understand the underlying mechanisms of TBS. Future studies in more advanced human neuron models such as (patient-specific) neuronal cultures derived from induced pluripotent stem cells (iPSC’s; Takahashi and Yamanaka, 2006) or cerebral organoids (Lancaster and Knoblich, 2014), could help improve our understanding of individual differences in responsiveness to stimulation protocols.

## Conclusion

The molecular mechanisms of rTMS remain largely undiscovered, and most of the evidence for plasticity effects following stimulation comes from animal models. In this study, we stimulated living *human* neurons (SH-SY5Y cells) with iTBS and cTBS protocols, and investigated changes in gene expression and morphology. We found evidence for a protocol specific increase in the expression of plasticity genes in the BDNF-TrkB pathway at 24 h following iTBS, relative to sham. In this human neuron model, we show the feasibility of studying rTMS effects *in vitro*, and we identify several gene expression changes that support iTBS-induced plasticity. These findings pave the way to develop more complex *in vitro* models, such as neuronal cultures from patient-derived iPSCs, in order to better examine the molecular effects of TBS, which in turn is necessary to further optimize the stimulation parameters for human rTMS.

## Data Availability Statement

The raw data supporting the conclusions of this manuscript is available through the following link: https://doi.org/10.34894/SP4AWN.

## Author Contributions

AT, ST, and GK contributed to conception and design of the study. AT performed the experiments and analysis. AT wrote the first draft of the manuscript. GK, ST, TG, TS, BR, and AS provided supervision and contributed significant input into the manuscript. All authors have read and approved the submitted version.

## Conflict of Interest

The authors declare that the research was conducted in the absence of any commercial or financial relationships that could be construed as a potential conflict of interest.
