# Supplementary material for: Transcranial Magnetic Stimulation-Induced Plasticity Mechanisms: TMS-Related Gene Expression and Morphology Changes in a Human Neuron-Like Cell Model
Source: Front Mol Neurosci. 2020 Oct 19;13:528396. doi: 10.3389/fnmol.2020.528396 (PMC7604533; doi:10.3389/fnmol.2020.528396)
Supplement: Supplementary file 1 [file Table_1.DOCX]

Supplementary Material

# Supplementary Data

## Immediate Time Point One-Way ANOVA

Each gene was analyzed separately, to test for an immediate effect of stimulation on gene expression. MAP2: F(14)=1,438, p=0.276, BCL2:F(14)= 0.488, p=0.625, EGR1: F(17)= 0.174, p=0.842, CREB1: F(17)=0. 659, p=0. 532, TUBB3: F(17)= 0.074, p=0.926, NTRK2: F(14)= 0.857, p=0.449.

## Differentiation analysis Gene expression

Independent samples student’s t-tests (2-tailed) were performed for each gene separately, comparing expression levels between undifferentiated and 10 day differentiated cells. MAP2: t(14)=-2.679, p=0.018, BCL2:t(14)=-2.769, p=0.017, EGR1: t(14)=-1.537, p=0.147, CREB1: t(14)=-1.389, p=0.186, TUBB3: t(14)=0.095, p=0.926, NTRK2: T(14)=-8.636, p<0.0001.

## **Effect of Stimulation on Housekeeping Genes**

There was no significant effect of Time or Condition, or Interaction, on any of the housekeeping genes used to normalize gene expression values for further analysis. Housekeeping genes used: TBP Condition(F(2,30)=0.685, p=0.513), Time(F(1,30)=0.001, p=0.977), Interaction(F(2,30)=0.587, p=0.563); GAPDH Condition(F(2,30)=0.091, p=0.913), Time(F1,30=0.111, p=0.741), Interaction(F(2,30)=0.058, p=0.944); PPiB Condition(F(2,30)=0.269, p=0.766), Time(F(1,30)=0.839, p=0.367), Interaction(F(2,30)=0.009, p=0.991). An average of these 3 genes was made, which was also not significant for Condition, Time or Interaction; Condition(F(2,30)=0.163, p=0.851), Time(F(1,30)=0.146, p=0.705), Interaction(F(2,30)=0.066, p=0.936).

## Power Analysis

A post-hoc power analysis was done using G*Power (3.1.9.7)

Test family: F tests

Statistical test: ANOVA: Fixed effects, omnibus, one-way

Type of power analysis: Post-Hoc: Compute achieved power-given α, sample size, and effect size

**For Condition:**

Effect size f=0.25

Α err prob=0.05

Total Sample size=21

Number of groups: 3

Output:

Noncentrality parameter λ= 1.3125

Critical F=3.555

Numerator df=2

Denominator df=18

Power (1-β err prob)=0.1429381

**For Time:**

Effect size f=0.25

Α err prob=0.05

Total Sample size=16

Number of groups: 2

Output:

Noncentrality parameter λ= 1.0000

Critical F=4.6001

Numerator df=1

Denominator df=14

Power (1-β err prob)=0.1540475

# Supplementary Figures and Tables

## Supplementary Figures

**
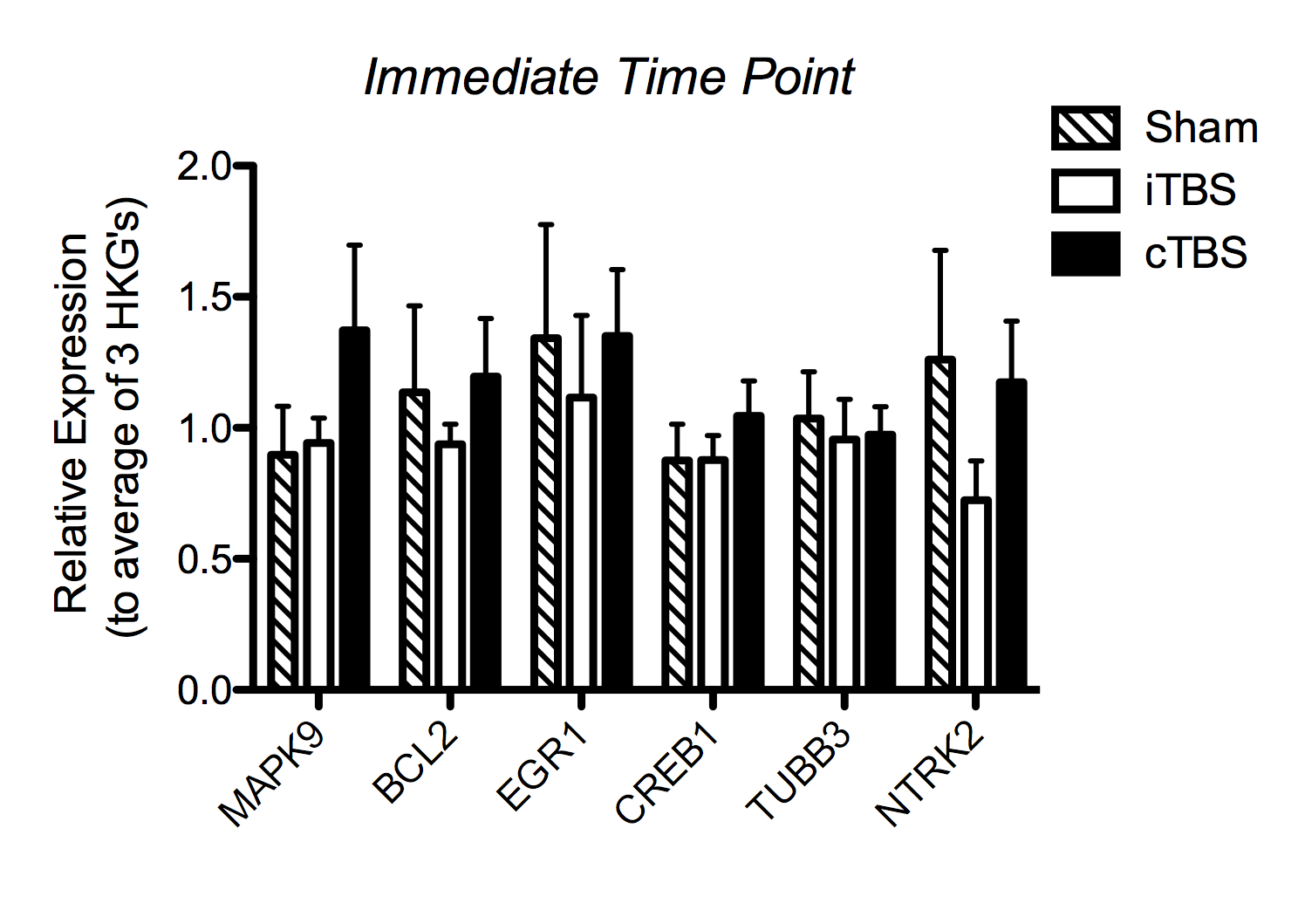
**

**Supplementary Figure 1.**Gene expression of all genes (*MAPK9, BCL2, EGR1, CREB1, TUBB3, NTRK2*) of interest in each of the conditions (cTBS, iTBS, sham) at the immediate time point (collected immediately after stimulation). None of the genes showed any significant effect of stimulation condition (P<0.05).


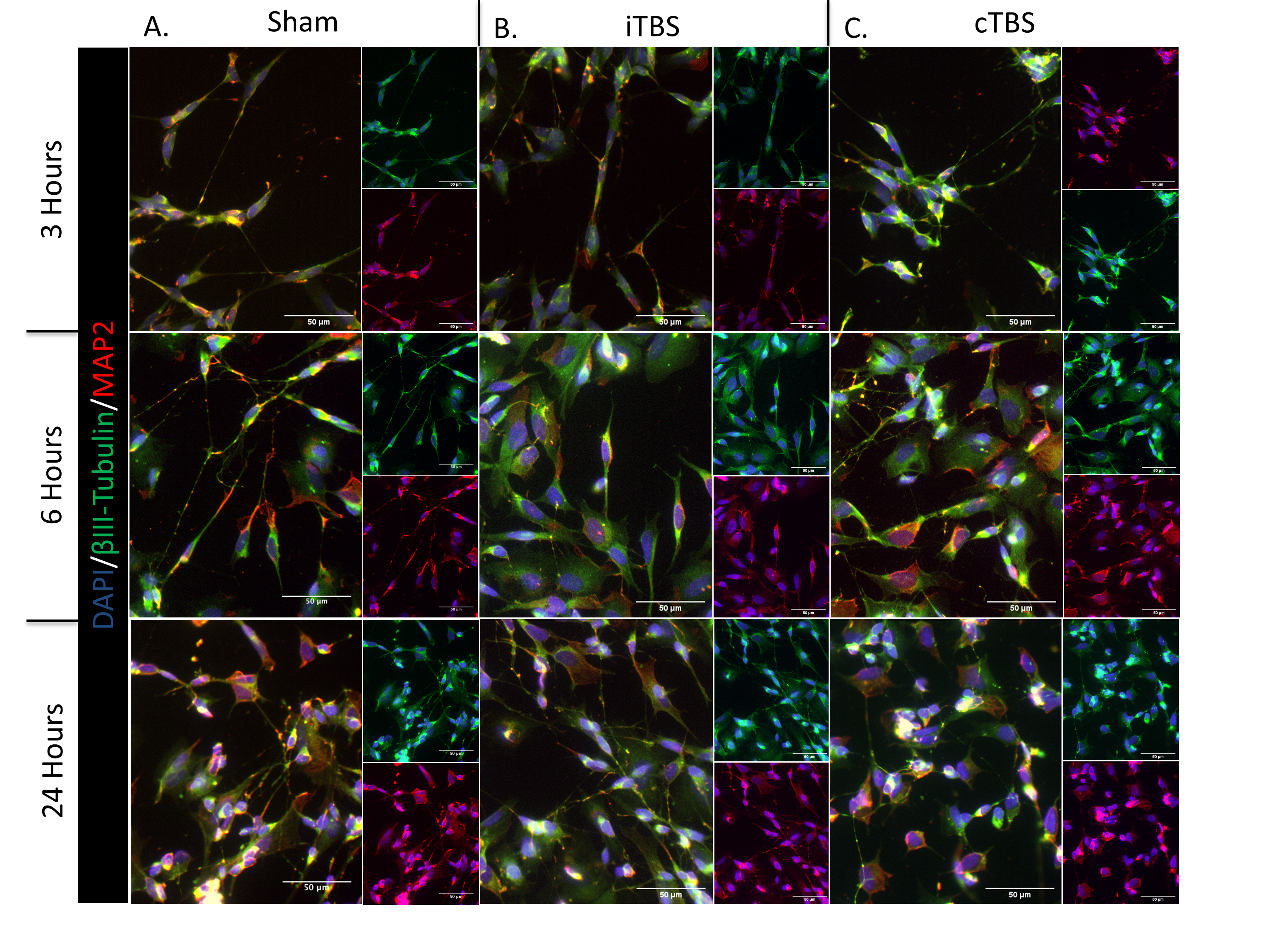


**Supplementary Figure 2.** Representative images of neuron morphology at each time point following each stimulation condition. DAPI (cell nucleus) in blue, βIII-Tubulin in green, and MAP2 in red. Each condition/time point image has 1 large merged image on the left (βIII-Tubulin and MAP2), and on the right 1 small βIII-Tubulin image, and 1 small MAP2 image. From Top to bottom, 3 hours, 6 hours, 24 hours. From left to right: **A.** Sham Stimulation, **B.** iTBS stimulation, **C.** cTBS stimulation. Scale Bar is 50 μM.

## Supplementary Tables

| **Condition** | **Time** | **Biological Replicates** | **Technical Replicates (per biological replicate)** | **Independent Experiments** |
| --- | --- | --- | --- | --- |
| cTBS | Immediate | 6 | 2 | 4 |
| iTBS | Immediate | 7 | 2 | 4 |
| Sham | Immediate | 5 | 2 | 4 |
| cTBS | 6h | 8 | 2 | 2 |
| iTBS | 6h | 7 | 2 | 2 |
| Sham | 6h | 6 | 2 | 2 |
| cTBS | 24h | 5 | 2 | 2 |
| iTBS | 24h | 4 | 2 | 2 |
| Sham | 24h | 6 | 2 | 2 |

## **Table S1**: qPCR sample collection.

| **Name** | **Fwd/Rev** | **Sequence 5’🡪3’** |
| --- | --- | --- |
| *NTRK2* | Forward | TGGATGCATATCGTGCTCCG |
| *NTRK2* | Reverse | GTGCTTGGTTCAGCTCTTGC |
| *BCL2* | Forward | ACATCGCCCTGTGGATGACT |
| *BCL2* | Reverse | CCGTACAGTTCCACAAAGGC |
| *MAPK9* | Forward | TGGGCTACAAAGAGAACGTTGA |
| *MAPK9* | Reverse | GTGCCTTGGAATATCACACAACC |
| *TUBB3* | Forward | GGGGCCTTTGGACATCTCTTC |
| *TUBB3* | Reverse | GTGTAGTGACCCTTGGCCC |
| *EGR1* | Forward | CCCCGACTACCTGTTTCCAC |
| *EGR1* | Reverse | GACAGAGGGGTTAGCGAAGG |
| *CREB1* | Forward | CCCCAGCACTTCCTACACAG |
| *CREB1* | Reverse | CTCGAGCTGCTTCCCTGTTC |
| *GAPDH* | Forward | CCAAATGCGTTGACTCCGA |
| *GAPDH* | Reverse | GCATCTTCTTTTGCGTCGC​ |
| *PPiB* | Forward | GTTTGAAGTTCTCATCGGGG |
| *PPiB* | Reverse | AAAACAGCAAATTCCATCGTG |
| *TBP* | Forward | TGCACAGGAGCCAAGAGTGAA |
| *TBP* | Reverse | CACATCACAGCTCCCCACCA |

**Table S2:** Primer sequences, 5’ to 3’ orientation.

| **Analysis Mode** | **Program Name** | **cycles** | Target (°C) | Acquisition Mode | Hold (hh:mm:ss) | Ramp Rate (°C/s) |
| --- | --- | --- | --- | --- | --- | --- |
| None | Pre Incubation | 1 | 95 | None | 0:10:00 | 4.8 |
| Quantification | Amplification | 45 | 95 | None | 0:00:10 | 4.8 |
|  |  |  | 60 | single | 0:00:45 | 2.5 |
| Melting Curves | Melting Curve | 1 | 95 | None | 0:00:15 | 4.8 |
|  |  |  | 60 | None | 0:00:30 | 2.5 |
|  |  |  | 97 | Continuous |  | 0.11 |
| None | Cooling | 1 | 60 | None | 0:00:10 | 2.5 |

**Table S3:** qPCR program.

| **Name** | **Company** | **Order number** | **Dilution** | **Marker of** | **Secondary**  **Antibody** |
| --- | --- | --- | --- | --- | --- |
| βIII-Tubulin | Cell Signaling | 5568S | 1:300 | Neurons;  Neurite outgrowth | donkey anti rabbit Alexa 488(Invitrogen, A-21206) |
| MAP2 | Sigma | M2320 | 1:300 | Neurons;  Axons | donkey anti mouse Alexa 594(Invitrogen, A-21203), |

**Table S4:** Secondary antibody information.

| **Wavelength** | **Marker of** | **Exposure Time** | **Color** |
| --- | --- | --- | --- |
| 350 | DAPI(nuclei) | 10ms | Blue |
| 488 | βIII-Tubulin | 500ms | Green |
| 594 | MAP2 | 300ms | Red |

**Table S5:** Fluorescence microscopy detection. Olympus BX51WI microscope and DSU spinning unit, 20X objective.

### Key Resources Table

| **REAGENT or RESOURCE** | | **SOURCE** | **IDENTIFIER** |
| --- | --- | --- | --- |
| **Chemicals, Peptides, and Recombinant Proteins** | | | |
| βIII-Tubulin Rabbit mAb | | Cell Signaling | Cat #5568S  RRID:AB_10694505 |
| Monoclonal Anti-MAP2 antibody produced in mouse | | Sigma-Aldrich | Cat # M2320  RRID:AB_609904 |
| Donkey Anti-Rabbit IgG Antibody | | Molecular Probes | Cat #A-21206  RRID:AB_141708 |
| Donkey Anti-Mouse IgG Antibody | | Molecular Probes | Cat #A-21203  RRID:AB_141633 |
| **Experimental Models: Cell Lines** | | | |
| Homo sapiens; SH-SY5Y | ATCC | | Cat# CRL-2266  RRID:CVCL_0019 |
| **Software and Algorithms** | | | |
| Fiji (ImageJ) | | Schindelin et al., 2012 | <http://fiji.sc>; RRID:SCR_002285 |
| Prism version 5.0 | | GraphPad Prism | [www.graphpad.com](http://www.graphpad.com);  RRID:SCR_002798 |
| Micro-Manager software | | Edelstein et al., 2014 | <http://micro-manager.org/>;  RRID:SCR_016865 |
